# Adherence to general medical checkup and cancer screening guidelines according to self-reported smoking status: Korea National Health and Nutrition Examination Survey (KNHANES) 2010–2012

**DOI:** 10.1371/journal.pone.0224224

**Published:** 2019-10-22

**Authors:** Eun Young Kim, Young Sup Shim, Young Saing Kim, Sang Pyo Lee, Ki Dong Ko, Won-Jun Choi

**Affiliations:** 1 Department of Radiology, Gil Medical Center, Gachon University College of Medicine, Incheon, South Korea; 2 Department of Information and Statistics, Korea National Open University, Seoul, South Korea; 3 Department of Internal Medicine, Gil Medical Center, Gachon University College of Medicine, Incheon, South Korea; 4 Department of Family Medicine, Gil Medical Center, Gachon University College of Medicine, Incheon, South Korea; 5 Department of Occupational and Environmental Medicine, Gil Medical Center, Gachon University College of Medicine, Incheon, South Korea; Medical University of South Carolina, UNITED STATES

## Abstract

**Objectives:**

The National Lung Screening Trial (NLST) revealed that low-dose computed tomography (LDCT) screening could reduce lung cancer mortality in heavy smokers. Lung screening with LDCT was implemented in July 2019 as part of the National Cancer Screening Program in Korea for heavy smokers who meet NLST criteria [smokers aged 55–74 years with 30 pack-years (PY) or more, excluding former smokers with more than 15 years since smoking cessation]. This study evaluated NLST-eligible heavy smokers’ adherence to general medical checkup and cancer screening guidelines.

**Methods:**

Using the Korea National Health and Nutrition Examination Survey (KNHANES) from 2010 to 2012, we compared adherence of Korean adults (55–74 years, n = 5,480) to general medical checkup and cancer (gastric, colorectal, breast, and cervical) screening guidelines according to self-reported smoking status. Smoking and PY data were available, but no data indicating when former smokers ceased smoking were available. Accordingly, smoking status was only classified as NLST (smokers with a history ≥ 30 PY) and non-NLST. Individuals who met NLST criteria were subdivided into current (NLST-current) and former smokers (NLST-former). Multivariable logistic regression was used to evaluate adherence to screening recommendations as a function of the study group (NLST-current, NLST-former, non-NLST) using possible covariates (sociodemographic factors, health-related behaviors, comorbidities, and self-reported health status).

**Results:**

Weighted prevalence of NLST-current was 9.7%, of NLST-former was 9.6%, and of non-NLST was 80.7%. Overall screening rates were 70.7% (medical checkup), 59.1% (stomach cancer), 58.1% (colorectal cancer), 59.1% (breast cancer), and 48.9% (cervical cancer). Adherence to colorectal cancer screening and medical checkup was lower in NLST-current than non-NLST (AOR 0.59; 95% CI 0.44–0.78 for colorectal cancer; AOR 0.70; 95% CI 0.52–0.95 for medical checkup). Screening practices for other cancers were not different.

**Conclusions:**

Current heavy smokers meeting NLST criteria were less likely to have colorectal cancer screening or general medical checkup. Understanding the screening practices of this target population might enable the development of more effective plans to implement lung screening and improve screening compliance for other cancers.

## Introduction

Lung cancer is one of the most common malignancies and a leading cause of cancer death among both men and women [1]. Lung cancer frequently presents at advanced stages, and the prognosis is poor. Given that the stage of the tumor has the greatest impact on lung cancer prognosis, early diagnosis is important. In 2011, the National Lung Screening Trial (NLST) showed that low-dose computed tomography (LDCT) screening led to a 20.3% reduction in lung cancer mortality and a 6.7% decrease in all-cause mortality [2]. The NLST selected participants with a high risk of lung cancer based on age and cumulative tobacco smoke exposure. Participants were required to be 55 to 74 years old and heavy smokers with a history of 30 pack-years (PY) or more, except for ex-smokers with more than 15 years since smoking cessation [2]. Based on this evidence, the National Cancer Information Center has been recommending lung cancer screening for people who meet the NLST criteria since 2015 in Korea [3].

Cigarette smoking is the leading preventable cause of death [4, 5] and is known to cause cancer in various organs, including the lungs [6, 7], colon [8–10], breast [11, 12], stomach [13, 14], and uterus/cervix [15]. Moreover, smokers tend to have unhealthy lifestyle behaviors [16–18] and to comply less with cancer screening guidelines than never-smokers [19–21]. Therefore, although smoking cessation remains the most important cancer prevention method, subjects who smoke may potentially receive the greatest benefit from improved cancer screening, which could detect occult diseases at earlier stages.

In Korea, organized screening for stomach, colorectal, breast, and cervical cancer has been provided at no or minimal cost by the government as part of the National Cancer Screening Program (NCSP) since 2001 [5]. Since July 2019, lung cancer screening with LDCT has been included in the NCSP, and the target population is heavy smokers who meet the NLST criteria [22]. Understanding the screening practice pattern for this target population is important for the development of strategic plans to implement LDCT screening as well as to improve screening compliance for other cancers.

This study evaluated adherence to general medical checkup and cancer screening recommendations in heavy smokers who met NLST criteria.

## Materials and methods

### Study participants

We used data collected during the fifth Korea National Health and Nutrition Examination Survey (KNHANES V), which was conducted by the Korea Centers for Disease Control and Prevention from January 2010 to December 2012. The KNHANES is a cross-sectional, nationally representative survey conducted to determine the health and nutritional status of the civilian, noninstitutionalized Korean population.

**Fig 1:**
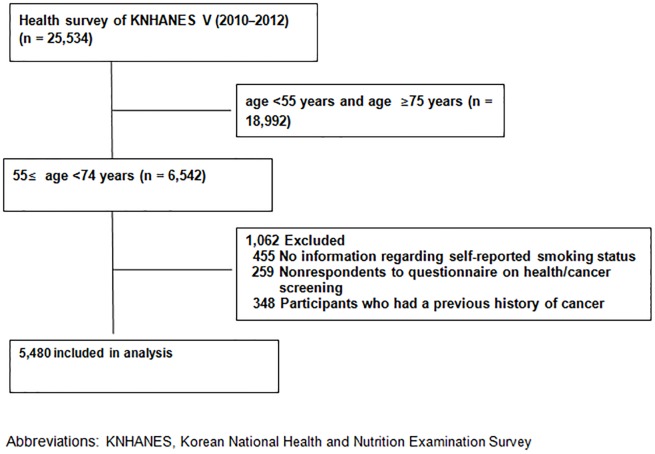
Flow diagram for selection of the study population.

The KNHANES is composed of a health questionnaire, nutrition survey, and health examination; participants were chosen by proportional allocation-systematic sampling with multistage stratification (by age, sex, and region). All data used in this study were fully anonymized prior to assessment. All procedures and terms and conditions of the survey have been complied with and were performed in accordance with the Declaration of Helsinki 7th version, and informed consent was obtained from all participants. The dataset and questionnaire is provided with guidelines for calculating a health-related index through the KCDC online site (https://knhanes.cdc.go.kr/knhanes/eng/index.do). Of the 25,534 participants, the final study population included a total of 5,480 individuals aged 55 to 74 years (Fig 1).

In the KNHANES, written informed consent was provided by every participant. The KNHANES survey was approved by the Institutional Review Board of the Korea Centers for Disease Control and Prevention (IRB No. 2010-02CON-21-C, 2011-02CON-06-C, 2012-01EXP-01-2C). Further ethical approval for the use of KNHANES data are not required because publicly available datasets were used in this study.

### Smoking status

In the survey, all participants were asked if they had smoked at least 100 cigarettes in their lifetime. Those who responded “yes” were further asked if they currently smoke cigarettes, which enabled individuals to be categorized as current smokers or former smokers. PY information was available for both current and ex-smokers; however, the KNHANES V did not indicate when ex-smokers quit smoking. Therefore, individuals were categorized into two groups according to their self-reported smoking status: NLST (smokers with a history of ≥30 PY) and non-NLST (never-smokers and smokers with a history of <30 PY). Individuals who met the NLST criteria were subdivided into current (NLST-current) and former smokers (NLST-former).

### Adherence to general medical checkups and cancer screening recommendations

Using the KNHANES for each cancer type, the participants were asked whether they had ever had a screening test, and if so, the length of time since the last test.

Adherence to general medical checkup guidelines was determined by asking participants if they had seen a doctor for a general medical checkup within the past two years. To define adherence to cancer screening guidelines, the NCSP [5] was used to determine age- and sex-appropriate cancer screening compliance; stomach cancer screening (endoscopy or upper gastrointestinal series) is recommended every 2 years in men and women 40 years of age or older; colorectal cancer screening (colonoscopy or barium enema) is recommended every 5 years in men and women 50 years of age or older; breast cancer screening (mammography) is recommended every 2 years in women 40 years of age or older; and cervical cancer screening (Papanicolaou (Pap) smear) is recommended every 2 years in women 30 years of age or older.

### Independent variables

Independent variables that have been associated with screening practices included sociodemographic variables [age [23, 24], sex, marital status [23, 25, 26], education level [23, 24, 27], household income [25, 26, 28], insurance status [23, 24, 27], and body mass index (BMI)], health-related lifestyle factors (drinking alcohol [28] and exercise), and health status (history of chronic diseases such as hypertension, diabetes, dyslipidemia, and perceived health status) [23, 24, 29].

Sociodemographic variables included current age (age of the respondents was categorized into five-year groups; 55 to 59, 60 to 64, 65 to 69, and 70 to 74) and marital status (unmarried, separated, widowed, and divorced participants were allocated ‘‘no spouse” status). Household income level was divided into national quartile groups (lowest quartile, second to third quartile, and highest quartile). Education level was categorized as less than elementary, middle/high school, and college or higher. National insurance status (national health insurance or medical aid), private health insurance (no, yes), and weight status (BMI <25 kg/m^2^ or BMI ≥25 kg/m^2^) were also included [30].

For health-related lifestyle factors, alcohol consumption was assessed using the Alcohol Use Disorders Identification Test (AUDIT, in which scores of ≥12 are heavy drinkers and <12 are not heavy drinkers) [31]. Routine exercisers were defined as people who performed at least low-intensity physical activity, which was defined as walking or commuting for >30 minutes more than three times per week.

Comorbidities include self-reported physician’s diagnoses of hypertension, diabetes, and dyslipidemia, and perceived health status is categorized as good, normal, and poor.

### Statistical analysis

Baseline characteristics are presented as percentages (± standard errors of percentages) for categorical variables and as estimated means (± standard errors of means) for continuous variables according to smoking status. Categorical variables and continuous variables were compared using the chi-square test and the Student’s t-test, respectively. The chi-square test was used to compare screening rates between subjects with different smoking statuses (NLST-current, NLST-former, non-NLST); *P* values < .016 were considered significant using Bonferroni’s method in case of multiple comparisons.

Using multivariable logistic regression, we calculated the odds ratio (OR) and 95% confidential interval (CI) for the probability of receiving screening as a function of the study group (NLST-current, NLST-former, non-NLST) and other covariates. Gradual modeling was used for adjusting potential covariates. Covariates in model 1 included sociodemographic factors, such as sex, age group, BMI, education level, marital status, insurance status, and private insurance; covariates in model 2 included sociodemographic factors and behavioral risk factors (alcohol consumption and exercise); and covariates in model 3 included sociodemographic factors, behavioral risk factors, and personal health status (comorbidities and perceived health status).

All analyses were adjusted for the complex survey design in the KNHANES using the complex sample analysis program in Predictive Analytics Software 18.0 (SPSS Inc., Chicago, Illinois, USA), and *P* values < .05 (two-sided) were considered significant.

## Results

### General characteristics of the study population

Characteristics of the study sample are shown in Table 1. Of the 5,480 participants (mean age, 63.1 ± 0.1 years; men, 48.0%), the weighted prevalence of NLST-current was 9.7% (unweighted n = 432), NLST-former was 9.6% (unweighted n = 489) and of non-NLST was 80.7% (unweighted n = 4,559).

**Table 1:** Characteristics of Korean adults (55–74 years) according to self-reported smoking status (n = 5,480).

Characteristics	Non-NLST (unweighted n = 4,559)	NLST-former (unweighted n = 489)	NLST-current (unweighted n = 432)	*P* value
Sex, male	36.1 (0.7)	98.0 (0.9)	97.3 (1.1)	< .001*
Age (mean, years)	63.2 (0.1)	63.7 (0.3)	61.6 (0.3)	< .001^†^
BMI (mean, years)	24.3 (0.1)	24.3 (0.2)	23.3 (0.2)	< .001^†^
Obesity (BMI ≥25 kg/m^2^)	38.7 (1.0)	39.8 (2.7)	25.7 (2.3)	.001*
Spouse, yes	80.7 (0.7)	90.8 (1.8)	90.8 (1.7)	< .001*
Household income				.846*
Lowest quartile	28.9 (1.0)	28.2 (2.4)	31.4 (2.6)	
2^nd^ and 3^rd^ quartile	51.2 (1.1)	50.3 (2.7)	49.4 (2.7)	
Highest quartile	19.9 (0.9)	21.4 (2.4)	19.2 (2.4)	
Education				< .001*
Less than elementary school	52.6 (1.1)	42.3 (2.9)	39.0 (3.0)	
Middle or high school	37.9 (1.0)	47.0 (2.8)	54.1 (3.0)	
College and above	9.5 (0.7)	10.7 (1.6)	6.9 (1.5)	
Insurance				.005*
National health insurance	97.5 (0.3)	94.6 (1.3)	96.5 (1.0)	
Medical aid	2.5 (0.3)	5.4 (1.3)	3.5 (1.0)	
Private Insurance, yes	59.3 (1.0)	56.9 (2.8)	55.0 (3.2)	.316*
Heavy alcohol drinker	15.2 (0.8)	36.3 (2.9)	49.3 (2.8)	< .001*
Routine exercise	46.8 (1.0)	49.9 (2.8)	44.4 (3.2)	.390*
HTN	41.0 (1.0)	45.0 (2.7)	29.5 (2.6)	< .001*
DM	14.0 (0.6)	19.0 (2.2)	23.1 (2.5)	< .001*
Hyperlipidemia	19.1 (0.8)	16.9 (2.2)	15.3 (2.3)	.217*
Perceived health status				.266*
Good to very good	28.6 (0.9)	28.6 (2.5)	24.4 (2.5)	
Normal	45.2 (1.0)	49.1 (2.8)	49.2 (2.8)	
Poor to very poor	26.2 (0.8)	22.3 (2.4)	26.4 (2.6)	

Values are weighted means (standard errors of means) or weighted percentages (standard errors of percentages).

*Pearson’s chi-squared test.

^†^Student’s t-test

Abbreviations: BMI, body mass index; HTN, hypertension; DM, diabetes mellitus

NLST-current and NLST-former had a higher proportion of male subjects than non-NLST (proportion of male subjects: 97.3% NLST-current, 98.0% NLST-former, 36.1% non-NLST, *p <* .001). Household income, private insurance, routine exercise, presence of hyperlipidemia, and perceived health status were not significantly different between subjects with different smoking status.

### General medical checkup and cancer screening practices

National health and cancer screening rates in NLST-current, NLST-former, and non-NLST are shown in Table 2. In Korean adults aged 55–74 years, the overall adherence to general medical checkup guidelines was 70.7%. The overall adherence to stomach cancer screening recommendations was 59.1%, colorectal cancer was 58.1%, breast cancer was 59.1%, and cervical cancer was 48.9%. In NLST-current, the adherence for general medical checkups, and for stomach, colorectal, breast, and cervical cancer screenings were 64.2%, 52.8%, 46.1%, 59.1%, and 48.9%, respectively. The screening rates for general medical checkups (*P* = .011; 62.4% in NLST-current vs. 71.6% in non-NLST) and colorectal cancer (*P* < .001; 46.1% in NLST-current vs. 59.1% in non-NLST) were significantly lower in NLST-current compared with non-NLST.

**Table 2:** Medical checkup and cancer screening rates among Korean adults aged 55–74 years according to self-reported smoking status.

	Non-NLST (unweighted n = 4,559)	NLST-former (unweighted n = 489)	NLST-current (unweighted n = 432)	Total (unweighted n = 5,480)	*P* value
General medical checkup in last 2 yrs	71.6 (0.9)	70.2 (2.6)	64.2 (3.1)	70.7 (0.9)	.038^b^
Stomach cancer screening in last 2 yrs	59.9 (1.0)	59.3 (2.9)	52.8 (3.1)	59.1 (0.9)	.075
Colorectal cancer screening in last 5 yrs	59.1 (1.0)	61.8 (2.8)	46.1 (2.9)	58.1 (1.0)	< .001^c^
Breast cancer screening in last 2 yrs^a^	59.2 (1.1)	74.4 (15.3)	40.5 (21.2)	59.1 (1.1)	.487
Cervical cancer screening in last 2 yrs^a^	48.9 (1.2)	74.7(15.3)	36.0 (21.7)	48.9 (1.2)	.341

^a^Females only (n = 3,164)

^b^*P* = .011 for NLST-current vs. non-NLST, *P* = .612 for NLST-former vs. non-NLST

^c^*P* < .001 for NLST-current vs. non-NLST, *P* = .362 for NLST-former vs. non-NLST

Crude and adjusted OR for medical checkups and cancer screenings according to smoking status are shown in Table 3. For general medical checkups, NLST-current had lower adherence than non-NLST in the unadjusted model (OR 0.71; 95% CI 0.55–0.93) and even after adjusting for covariates (AOR 0.70; 95% CI 0.52–0.95 for model 3). NLST-current had a lower adherence to colorectal cancer screening recommendations than non-NLST even after adjusting for sociodemographic factors, health-related behaviors, and comorbidities (AOR 0.59; 95% CI 0.44–0.78). However, NLST-former showed no significant difference in adherence to general medical checkups and colorectal cancer screening. Furthermore, adherence to other cancer (gastric, breast, and cervical cancer) screenings was not different according to the self-reported smoking status.

**Table 3:** Unadjusted and adjusted odds ratio (95% confidence interval) for medical checkup and cancer screening practices according to self-reported smoking status.

	Unadjusted OR (95% CI)	Model 1 AOR (95% CI)	Model 2 AOR (95% CI)	Model 3 AOR (95% CI)
**General medical checkup in last 2 yrs**				
Non-NLST	Reference	Reference	Reference	Reference
NLST-former	0.94 (0.72–1.21)	0.85 (0.63–1.15)	0.86 (0.63–1.17)	0.84 (0.61–1.15)
NLST-current	0.71 (0.55–0.93)	0.67 (0.50–0.90)	0.69 (0.51–0.93)	0.70 (0.52–0.95)
**Stomach cancer screening in last 2 yrs**				
Non-NLST	Reference	Reference	Reference	Reference
NLST-former	0.94 (0.77–1.25)	0.94 (0.71–1.25)	0.99 (0.73–1.32)	0.97 (0.72–1.31)
NLST-current	0.75 (0.59–0.96)	0.74 (0.57–0.989)	0.76 (0.57–1.01)	0.77 (0.57–1.03)
**Colorectal cancer screening in last 5 yrs**				
Non-NLST	Reference	Reference	Reference	Reference
NLST-former	1.12 (0.88–1.42)	1.10 (0.83–1.46)	1.11 (0.83–1.49)	1.09 (0.81–1.46)
NLST-current	0.59 (0.47–0.75)	0.59 (0.45–0.77)	0.59(0.45–0.79)	0.59 (0.44–0.78)
**Breast cancer screening in last 2 yrs**^a^				
Non-NLST	Reference	Reference	Reference	Reference
NLST-former	2.01 (0.41–9.84)	4.95 (0.87–28.23)	2.17 (0.25–18.93)	2.35 (0.29–19.36)
NLST-current	0.47 (0.08–2.65)	0.51 (0.12–2.14)	0.48 (0.11–2.04)	0.49 (0.12–2.05)
**Cervical cancer screening in last 2 yrs**^a^				
Non-NLST	Reference	Reference	Reference	Reference
NLST-former	3.04 (0.62–14.96)	7.59 (1.19–48.27)	3.26 (0.36–29.28)	3.87 (0.42–35.24)
NLST-current	0.59 (0.09–3.76)	0.59 (0.13–2.74)	0.62 (0.13–2.99)	0.68 (0.15–3.20)

^a^Females only (n = 3,164)

Abbreviations: (A)OR, (adjusted) odds ratio, CI, confidence interval.

Model 1 was adjusted for sociodemographic factors such as sex, age group, obesity, education level, marital status, income level, insurance status, and private insurance. Model 2 was adjusted for sociodemographic factors and behavioral risk factors (alcohol consumption and exercise). Model 3 was adjusted for sociodemographic factors, behavioral risk factors, and personal health status (history of chronic disease and perceived health status).

## Discussion

Lung cancer is one of the most common malignancies and a leading cause of cancer deaths in men and women [1]. NLST, the randomized controlled trial, showed a reduction in lung cancer mortality (20.3%) and in all-cause mortality (6.7%) with LDCT screening in comparison with subjects who underwent chest radiography [2]. NLST defined participants with a high risk of lung cancer based on age (55–74 years) and cumulative tobacco smoke exposure (heavy smokers with a history of 30 pack-years (PY) or more, except for ex-smokers with more than 15 years since smoking cessation) [2]. Based on this evidence, the United States Preventive Service Task Force published recommendations encouraging annual lung screenings for individuals at high risk for lung cancer in March 2014. In Korea, lung cancer screening has commonly been conducted as a part of opportunistic screening programs, based on physician recommendations and individual preferences. Recently, the Korean government implemented lung screening with LDCT for heavy smokers who met NLST criteria as part of NSCP.

Although it is known that smokers tend to have unhealthy behaviors [16–18] and are less compliance with screening guidelines than non-smokers [19–21], no study has examined adherence to medical checkup guidelines and national cancer screening recommendations specific to heavy smokers in Korea, who are the target population for lung screening. In the present study, NLST-current had lower adherence to general medical checkup guidelines and colorectal cancer screening recommendations compared with non-NLST, even after adjusting for covariates. NLST-former was no different from non-NLST in the adherence to general medical checkup and colorectal cancer screening. These findings are similar to a recent study [32] that showed large differences in health care seeking practices between current and former smokers. Current smokers are less likely to screen for breast and colorectal cancer compared to never and former smokers [32]. Further research is needed to identify barriers to screening among current smokers, with the goal of increasing acceptance and uptake of cancer screening among this population at a high risk for cancer. Current heavy smokers’ low adherence to general medical checkup and colorectal cancer screening guidelines suggests that healthcare workers should also encourage these screenings in addition to lung screening.

This study has several limitations. First, defining smokers based on self-reporting is regarded as reliable in population-based surveys in Western populations [33]. However, several studies reported an underestimation of the true number of smokers in Asian populations [34]. Furthermore, smoking rates in women are reported as very low in East Asian countries, and under-reporting of hidden female smokers is an emerging issue. Furthermore, the KNHANES V did not include valid information regarding when former smokers quit smoking. Therefore, a slight disjoint of group definitions according to smoking status in the present study (NLST-current, NLST-former, non-NLST) could not be avoided. NLST-current and NLST-former are not identical in the target population for lung screening (i.e., NLST-current are the target population for lung screening but NLST-former are not if they quit smoking for more than 15 years), and non-NLST are not identical to never-smokers (non-NLST include smokers less than 30 PY, as well as never-smokers). Second, response bias could have been introduced when participants were asked questions about lifestyle habits, history of recent medical checkups, and cancer screening practices. This survey explored whether participants were screened in past years, and there is a potential for recall bias. Third, expecting LDCT adherence has limitations even with a full understanding of the adherence patterns associated with medical check-ups and other cancer screening practices. The LDCT examination has distinct characteristics from other screening methods. It is a less invasive and painful procedure compared to endoscopy, colonoscopy, or Pap smears. However, some participants might avoid lung screening due to the possible harms of LDCT examination (i.e., potential for radiation-induced carcinogenesis, high false-positivity rates, and overdiagnosis issue) [35].

In conclusion, current heavy smokers who meet NLST criteria were less likely to be screened for a general medical checkup and colorectal cancer; however, no difference was observed in adherence to general medical checkups and other cancer screenings. Understanding the screening practice patterns for this target population that is subject to lung screening recommendations might enable the development of more effective plans to implement lung screening as well as to improve screening compliance for other cancers.
